# The role of the immune system in progressive multifocal leukoencephalopathy: a comparative analysis of two cases following autologous and allogeneic hematopoietic stem cell transplantation

**DOI:** 10.1007/s00277-025-06258-5

**Published:** 2025-05-05

**Authors:** Biancamaria Mandelli, Roberta Mazzarella, Andrea Corbingi, Elettra Ortu La Barbera, Salvatore Perrone, David Fanciullo, Martina Lorenzon, Giada Pacitto, Natalia Cenfra, Sergio Mecarocci, Sofia Asioli, Alessandro Pulsoni

**Affiliations:** 1 Hematology, Department of Translational and Precision Medicine, Az. Policlinico Umberto I-Sapienza University, Rome, Italy; 2 Department of Hematology, Polo Universitario Pontino, S.M. Goretti Hospital, Latina, Italy; 3 Department of Biomedical and Neuromotor Sciences (DIBINEM)-Surgical Pathology Section, Alma Mater Studiorum - University of Bologna, Bologna, Italy

**Keywords:** Progressive Multifocal Leucoencephalopathy, Bone marrow transplantation, Acute myeloid leukemia, Diffuse large B cell lymphoma

## Abstract

Progressive multifocal leukoencephalopathy (PML) is a rare, subacute demyelinating disorder of the central nervous system (CNS) caused by the JCV. In immunosuppressed hosts, PML is caused by reactivation of a latent infection rather than primary exposure. Hematological patients, particularly post-transplant, presenting with worsening neurological symptoms should promptly consider PML in the differential diagnosis. The rarity of PML after autologous or allogeneic hematopoietic stem cell transplantation (HSCT) and the absence of a universally effective therapy represents a clinical challenge. Here, we present two cases of PML developed after autologous and allogeneic HSCT, with completely different outcomes dependent on the patients’ clinical backgrounds and the level of immune system competence which is the key factor in determining either the onset or viral clearance of the infection.

## Introduction

Progressive multifocal leukoencephalopathy (PML) is a rare demyelinating disorder of the central nervous system (CNS) caused by the JC human polyomavirus (JCV) [1, 2].

Immunosuppression is the primary key factor for viral reactivation and the onset of infection. Historically, a peak incidence was observed at the onset of the HIV epidemic, with numbers significantly decreasing following the introduction of highly active antiretroviral therapy [1, 2].

The epidemiology has undergone notable changes, particularly due to the introduction of monoclonal antibodies and cellular therapies, shifting primarily to the hematological, neurological and rheumatological settings.

In particular, the onset of progressively worsening neurological symptoms in the post-transplant setting should promptly consider PML in the differential diagnosis.

Herein, we present two cases of PML following autologous and allogeneic hematopoietic stem cell transplantation (HSCT) [3].

### Case 1

A 56-years-old man with diffuse large B-cell lymphoma underwent first-line chemotherapy with Rituximab, Cyclophosphamide, Vincristine, and Doxorubicin (R-CHOP) in 2017.

Following a relapse in 2023, he received high-dose salvage chemotherapy with Rituximab, Dexamethasone, Cisplatin, Cytarabine (R-DHAP) and subsequently underwent autologous HSCT.

Five months after, he developed neurological symptoms including dysphonia, dysarthria, and right upper limb weakness.

**Fig. 1:**
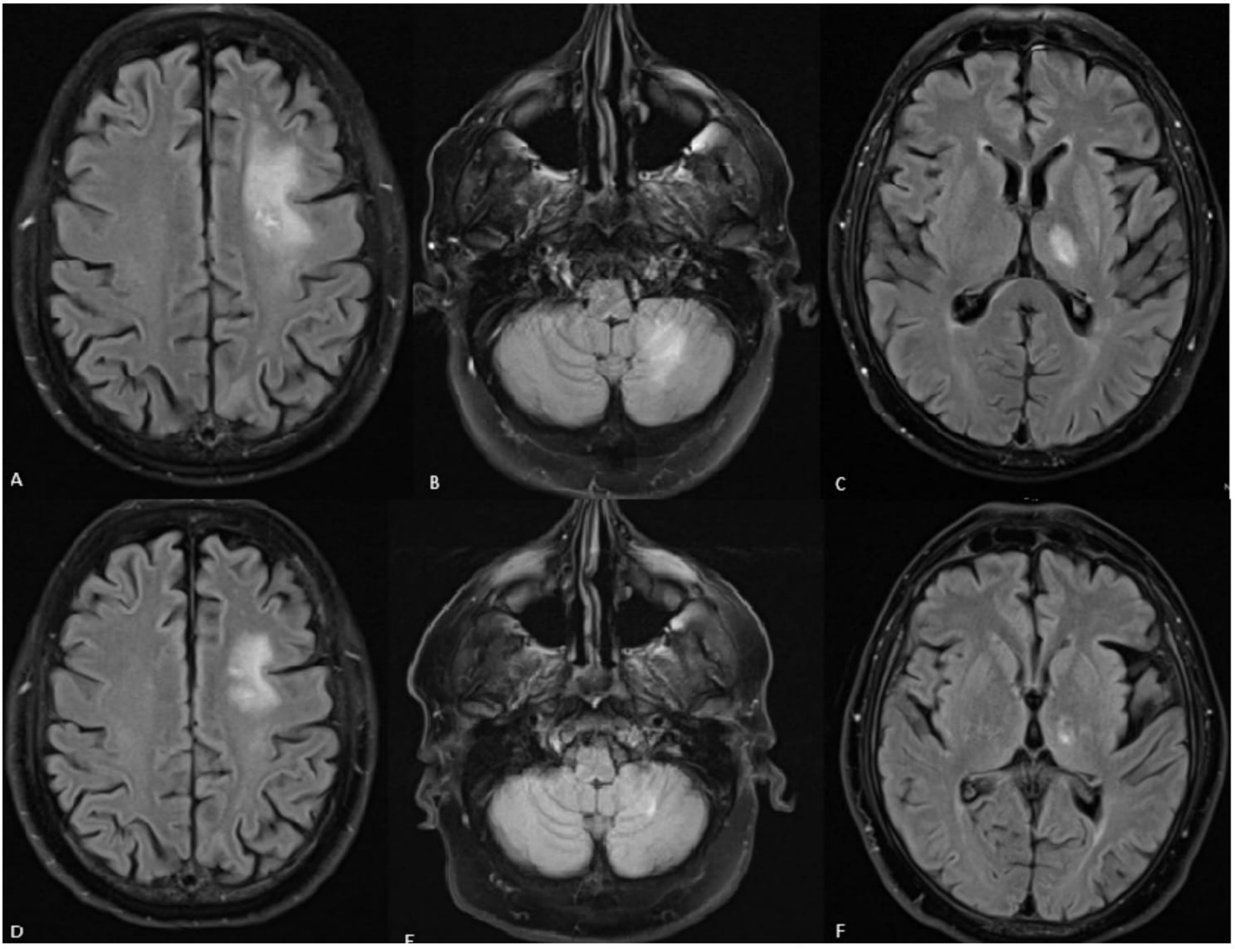
Brain MRI findings in *case n.1*, FLAIR sequencing. **A-B-C**: baseline sequences showing hyperintensity in the left fronto-parietal area, left cerebellar region and left thalamus respectively. **D-E-F**: sequences obtained after 2 weeks of steroid treatment revealed significant improvement and a reduction in lesion size

Brain magnetic resonance imaging (MRI) revealed hyperintense signal alterations in T_2_w and FLAIR sequencing in the left fronto-parietal area, left cerebellar region and left thalamus with vasogenic edema and heterogeneous enhancement in the central area after paramagnetic contrast agent (Fig. 1). After ruling out a recurrence of systemic lymphoma with a PET/CT scan, a diagnostic lumbar puncture (LP) was performed. Cerebrospinal fluid (CSF) analysis revealed no evidence of lymphoma via flow cytometry and showed elevated proteins at 55 mg/dL (normal range 20–30 mg/dL).

Polymerase chain reaction (PCR) testing for JCV, Epstein-Barr Virus (EBV), Herpes Simplex Virus 1–2 (HSV 1–2), Varicella Zoster Virus (VZV), Enterovirus, Toxoplasma, and Measles yielded negative results as well as bacteria and fungi tests.

In the absence of any diagnostic clues for relapsed lymphoma or infection, a stereotactic brain biopsy was performed to achieve a diagnosis. Histological examination revealed areas of demyelination associated with perivascular inflammatory lymphocytic infiltration, astrocytic and glial activation. Immunohistochemistry demonstrated a predominant T-lymphocyte infiltration, especially by CD8 + lymphocytes.

The clinical case was evaluated by a multidisciplinary team. Considering the clinical presentation, MRI findings and histological results the diagnosis appeared to be consistent with a vasculitic reaction.

**Fig. 2:**
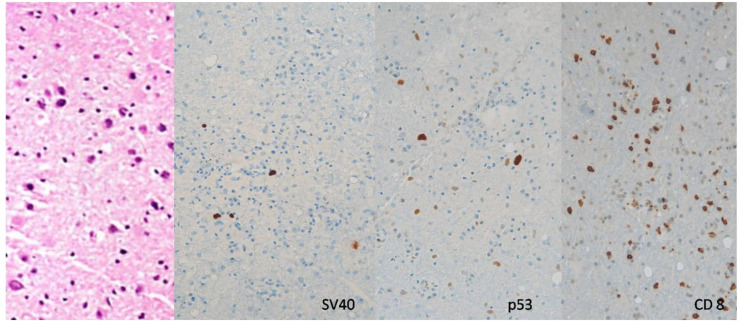
Case n.1 Immunohistochemistry of brain tissue showing positivity for SV40, p53 and CD8 infiltrates

Meanwhile, further investigations conducted at a specialized histopathology center demonstrated the presence of inclusion bodies suggestive of viral infection. Immunohistochemistry analysis using anti-CMV and anti-HSV antibodies was negative but tested positive for anti-SV40 and anti-P53 antibodies, and confirming the detection of JCV-DNA (Fig. 2). Based on these findings and MRI imaging, a conclusive histological examination confirmed CNS-Immune Reconstitution Inflammatory Syndrome (IRIS) in PML.

Therefore, one month after the onset of symptoms, methylprednisolone 1000 mg was administered for three days, followed by a reduction to 1 mg/kg for three further days. A gradual tapering was subsequently implemented over a two-month period until complete withdrawal while the patient experienced a marked improvement of brain lesions, ultimately leading to their resolution over a three-month period of MRI monitoring (Fig. 1) [4].

### Case 2

A 56-years-old woman with acute myeloid leukemia (AML) underwent allogeneic HSCT from HLA-identical sibling in October 2022. The conditioning regimen included thiotepa, busulfan, and fludarabine. Cyclosporine and methotrexate were given as Graft-Versus-Host Disease (GVHD) prophylaxis and discontinued 6 months after.

**Fig. 3:**
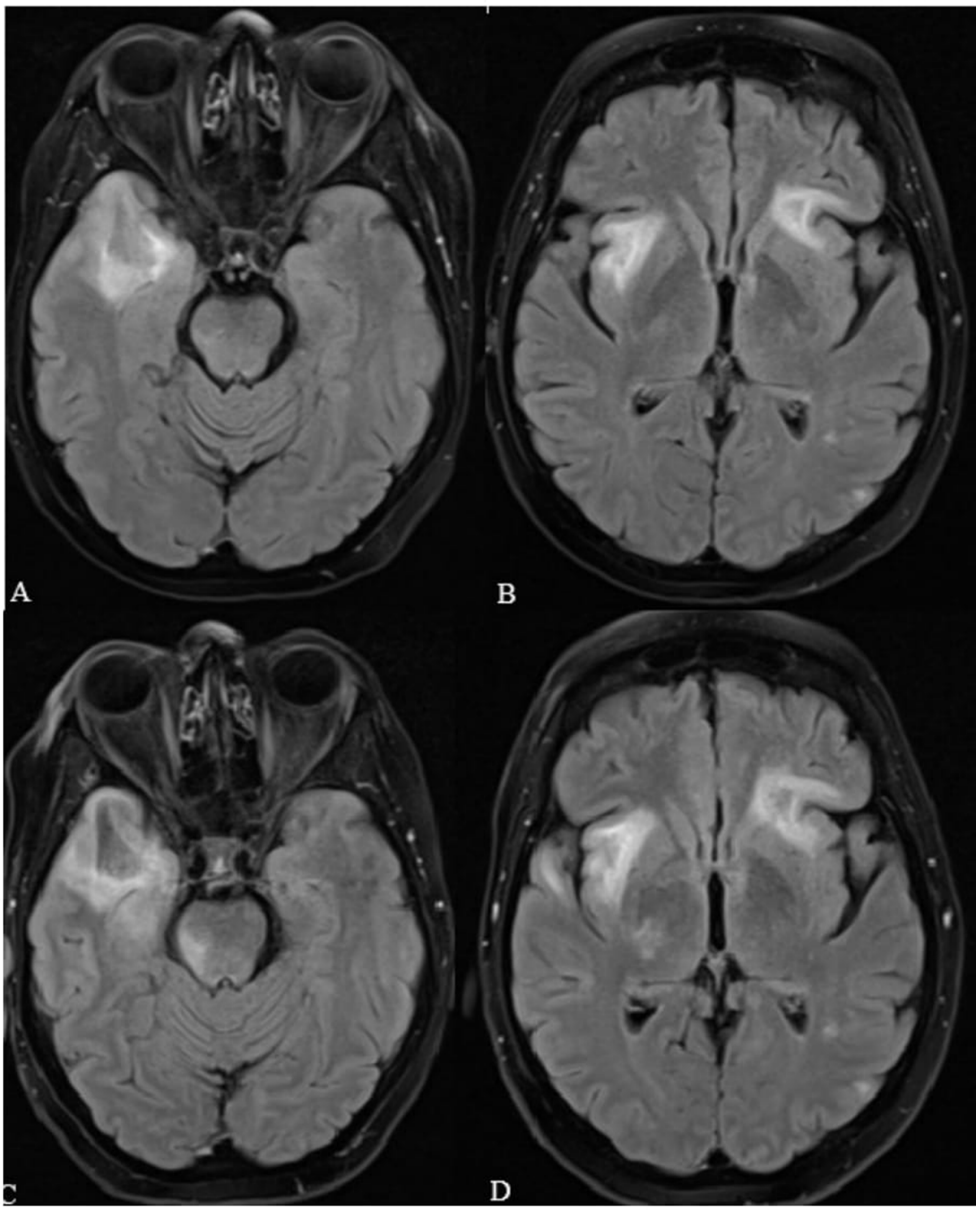
Brain MRI findings in *case n.2*, FLAIR sequencing. **A-B**: baseline sequences showing hyperintensity of the right temporal lobe, bilateral insular region and left frontal lobe. **C-D**: sequences obtained after 20 days of clinical worsening and confirming MRI progression

At 9 months post-HSCT, the patient developed cutaneous chronic GVHD and underwent steroid therapy. Shortly thereafter, she developed mild neurological symptoms which gradually worsened, including limbs weakness, dysarthria, and agnosia. Brain MRI revealed hyperintense lesions in T_2_w and FLAIR sequencing involving the subcortical white matter of the right temporal lobe, bilateral insular regions, left frontal lobe, right corticospinal tract and the right pontine area without any enhancement after paramagnetic contrast agent, highly suspicious for PML infection (Fig. 3).

A diagnostic LP was performed and CSF analysis showed elevated protein at 52 mg/dL and confirming JCV-DNA positivity (2495 copies/mL).

Although no specific therapy has been approved for PML, treatment with mirtazapine (30 mg daily) and cidofovir (5 mg/kg weekly, combined with probenecid) was administered but failed to produce any neurological improvement.

Given the limited efficacy of these treatments, the main purpose was to enhance the immune response by collecting lymphocytes from her HLA-identical donor and send them to a facility for in vitro expansion, in order to obtain anti-JCV lymphocytes capable of counteracting PML and eradicating JCV infection.

Despite the efforts, they proved to be unsuccessful since the patient’s neurological condition worsened, as confirmed by an MRI performed 20 days after the previous scan (Fig. 3), ultimately leading to her death two weeks after.

## Discussion

PML may manifest as a rare complication of JCV infection with insidious onset and no proven effective therapy.

In this report, we described two contrasting cases of PML infection. The clinical presentation varies widely, encompassing a spectrum of symptoms that depend on the severity of the infection and the host’s immune status.

Anti-JCV antibody activity is clinically important but not sufficient on its own. Indeed T-cell activity is necessary to prevent JCV reactivation and proliferation, thereby halting viral spread [5].

A relatively preserved immune system may result in an inflammatory reaction that cause immune-mediated damage, leading to PML-IRIS and possibly to the resolution of the infection [4].

Conversely, severe immunosuppression with a marked reduction in the antiviral activity of the immune system results in the classic presentation of PML, which is progressive and fatal. Between these extremes, there is a wide range of clinical presentations depending on the balance between immune system functionality and infection severity.

On one hand, in ***case 1***, the patient developed JCV infection due to reactivation in a post-transplant immunosuppressed state with prior anti-CD20 and chemotherapy exposure. JCV-DNA in CSF tested negative and brain biopsy has been the essential tool which led to diagnose PML infection.

The resolution of PML symptoms correlates with immunological recovery, as evidenced by normalization of T-CD4 + lymphocytes count (690/mm³) and a marked increase in T-CD8 + lymphocytes count (4200/mm^3^), which led to a rebound inflammatory reaction against brain infection evidenced by MRI findings which, in turn, resulted in viral clearance.

Steroid treatment played a key role in modulating the intensity of the immune response against PML infection and thereby contributing to its resolution [4].

On the other hand, in ***case 2***, the patient underwent allogeneic HSCT, thus with a much deeper immunological suppression, supported by previous conditioning regimen, steroid treatment and an unbalanced T-cell count (CD4 + 97/mm³, CD8 + 730/mm^3^) which led to a thriving PML infection and a rapid progressive disease.

The management of PML involves triggering a CNS response against brain JCV infection by correcting immune deficiencies and restoring immune function. A reduction in JCV-DNA levels in the CSF correlates with improved outcomes, as it reflects better viral control and immune clearance as well as prolonged T cell impairment is a negative prognostic factor. In such cases, experimental immunotherapies like PD-1 inhibitors, anti-JCV lymphocytes or clinical trials may be useful with a benefit provided which need to be assessed [4, 6].

Conversely, excessive immune responses, particularly with a large influx of T cells, can lead to CNS-IRIS, exacerbating symptoms and causing further brain damage. Thus, PML treatment requires balancing immune restoration to clear the virus while minimizing harmful inflammatory responses [4].

## Conclusion

PML following autologous or allogeneic HSCT is a rare and fatal complication. Clinical suspicion should always be considered when suggestive neurological symptoms arise, even when JCV-DNA testing is negative in the CSF, necessitating the consideration of a brain biopsy.

Treatment strategies for PML currently focus on enhancing JCV-specific T cell responses while preventing IRIS. Despite ongoing research and the exploration of various antiviral agents over the past decades, outcomes have generally been disappointing. Recent advancements include therapies aimed at restoring JCV-specific immunity or managing IRIS, although their effectiveness remains uncertain. Further studies are necessary to elucidate the optimal approach to manage this challenging condition especially in hematological patients.

## Data Availability

No datasets were generated or analysed during the current study.
